# Comparative Screening Analytic Methods for Elderly of Blood Methylmercury Concentration between Two Analytical Institutions

**DOI:** 10.1155/2018/2509413

**Published:** 2018-06-26

**Authors:** Byoung-Gwon Kim, Young-Seoub Hong, Koichi Haraguchi, Mineshi Sakomoto, Hyoun-Ju Lim, Jeong-Wook Seo, Yu-Mi Kim

**Affiliations:** ^1^Department of Preventive Medicine, College of Medicine, Dong-A University, Busan, Republic of Korea; ^2^Heavy Metal Exposure Environmental Health Center, Dong-A University, Busan, Republic of Korea; ^3^National Institute for Minamata Disease, Department of International Affairs and Research, Kumamoto, Japan; ^4^National Institute for Minamata Disease, Department of Environmental Science and Epidemiology, Kumamoto, Japan

## Abstract

Methylmercury is widely known to be a toxic substance in the human, especially a nervous system. However, it is difficult to accurately measure the amount of methylmercury in blood, and the form of methylmercury is variously presented. The purpose of study was to compare the total mercury and methylmercury measurements techniques and detection levels between analytical institutions in two countries using the same elderly human blood samples. Total mercury using gold amalgamation direct mercury analysis method (both) and methylmercury using the dithizone extraction and gas chromatography-electron capture detector (GC-ECD) method (N Lab in Japan) and the cold vapor atomic fluorescence spectrophotometer (CVAFS) method (D Lab in Korea) were measured in 47 subjects who agreed to participate in this study. Total mercury concentrations in both analytical laboratories were observed at similar levels (9.4 versus 9.5 ug/kg, p=0.898) and the distribution was highly correlated. However, the concentration of methylmercury showed some difference between two laboratories (9.1 versus 8.6 ug/kg, p<0.001). Due to different recovery rates by different analytical methods, it is assumed that the methyl/total mercury ratio in N lab in Japan was higher than D lab in Korea (96.8 versus 90.4%, p<0.001). The GC-ECD was more sensitive method than CVAFS in methylmercury analytic techniques.

## 1. Introduction

Mercury is present in various forms of elemental mercury (Hg_0_), inorganic mercury (HgCl_2_), and organic mercury (CH_3_HgCl) and exhibits various human toxicities according to the properties [1]. Organic mercury, especially methylmercury, caused a poisoning of fatal neurotoxicity [2–5]. Methylmercury at high doses is extremely well documented as a human neurotoxin, with effects mainly on the motor and sensory systems, especially in the area of sensory-motor integration. As with all chemicals, the amount of exposure and susceptibility of the host determine the effects [6, 7]. But nervous system effects in elderly have been used in establishing limits aimed at protecting the public's health [8–10]. Methylmercury is highly absorbed by humans (> 95% of the mercury ingested is absorbed by the body), and the fraction absorbed seems to be independent of the type of food [11–13].

Most of the current clinical studies on mercury exposure to elderly focus on measuring total mercury concentrations. Analysis on species of mercury, however, provides valuable information on possible contamination paths as well as mercury species distributions among different populations. Blood is usually analyzed to assess human mercury exposure [6]. Mercury species analysis, especially in the blood, can provide information about mercury sources. It is important to develop efficient tools to monitor human exposure to mercury, particularly species analysis [14, 15]. However, the problem of species separation analysis is that the matrix-dependent alkylation and dealkylation reactions can occur in the sample preparation and separation stages, leading to misjudgment. In the case of total blood mercury concentration analysis method, it was established to some extent, but a variety of analysis of methyl mercury was still proposed.

The gas chromatography-electron capture detector (GC-ECD) method and the cold vapor atomic fluorescence spectrophotometer (CVAFS) method are suggested for the analysis of methylmercury, and there are few studies that directly compare each method. Therefore, the authors conducted a comparative analysis of total mercury and methylmercury concentration using the same human sample in the analysis institutes of two countries using each analysis method.

## 2. Materials and Methods

### 2.1. Study Subjects and Sample Collection

The subjects of this study were participated from the campaign for the analysis of heavy metals in the local elderly residents' blood samples in 2015. The subjects agreed to participate in the study and completed the consent form. The subjects of the study were 30 males and 17 females. We collected the sex and age data for further analysis.

### 2.2. Metal Analysis

N laboratory in Japan analyzed total mercury in blood samples by a thermal decomposition amalgamation AAS (MA-3000, Nippon Instruments Corp., Japan). And the methylmercury concentrations were determined using the dithizone extraction and gas chromatography-electron capture detector (7890b, Agilent Technologies, USA) method [14]. D laboratory in Korea analyzed total mercury in blood samples by a gold amalgamation direct mercury analyzer (NIC-3000, Nippon Instruments Corp., Japan). And the methylmercury concentrations were determined using the cold vapor atomic fluorescence spectrophotometer (MERX, Brooks Rand Co., USA) method [16]. The analytical conditions of instruments and recovery test result by instruments were described in Table 1.

### 2.3. Statistical Analysis

This study conducted mean comparison analysis by sex group between two analysis laboratories. The significance level was 5% (p<0.05) in each test, and STATA/SE 12.0 (StataCorp., College Station, TX, USA) was used in all the statistical analyses.

### 2.4. Ethics

The protocol of this study was reviewed and approved by the Institutional Review Board of the Dong-A university hospital (IRB No. 13-010). Written informed consent was provided by all of the participants.

## 3. Results

### 3.1. Comparison of Total Mercury Concentration

**Figure 1:**
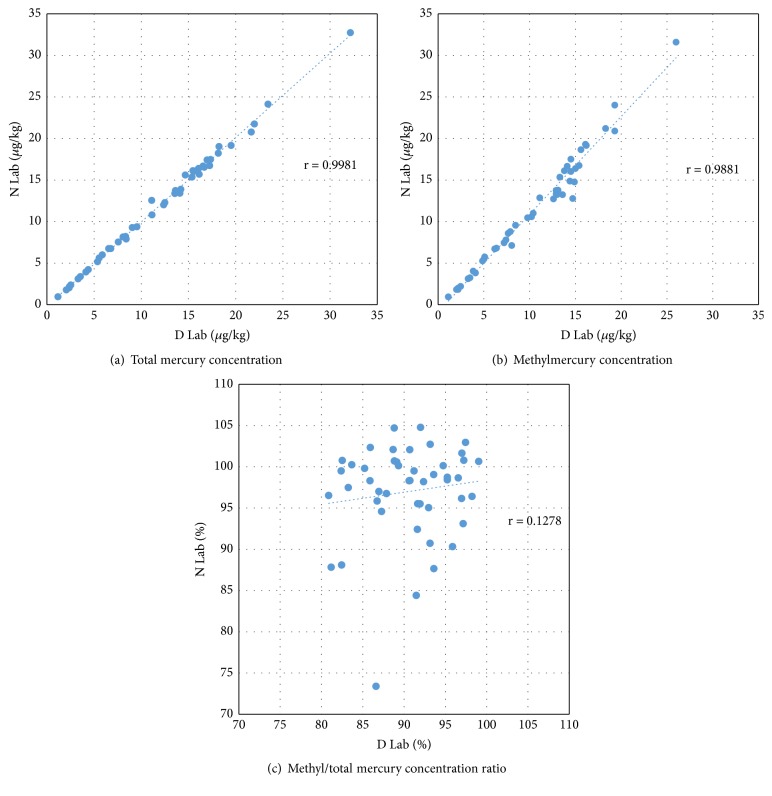
Distribution and correlation coefficient (r value) of (a) total mercury concentration, (b) methylmercury concentration, and (c) methyl/total mercury concentration ratio between D Lab in Korea and N Lab in Japan.

The subjects of the study were 30 males and 17 females. The average age was 58.6 ± 1.4 years, 60.8 ± 1.6 years for men and 54.6 ± 2.7 years for women. Total mercury concentrations of all subjects in D laboratory were 9.5 ug/kg and that by gender were 9.0 ug/kg for male and 10.5 ug/kg for female. Total mercury concentration of N Laboratory results by gender showed that female was higher than male in 9.4 ug/kg for all subjects, 8.8 ug/kg for male and 10.4 ug/kg for female, and correlation coefficient for all subjects was 0.9981 (Figure 1(a)). So total mercury concentrations of both laboratory were similar (p = 0.898) (Table 2).

### 3.2. Comparison of Methylmercury Concentration

Methylmercury concentration in D laboratory was found to be 8.6 ug/kg for all subjects and 8.2 ug/kg for male and 9.4 ug/kg for female. N laboratory results showed that female was higher than male at 9.1 ug/kg for all, 9.9 ug/kg for female, and 8.6 ug/kg for male (Table 2) and correlation coefficient of all subjects was 0.9881 (Figure 1(b)).

### 3.3. Comparison of Methyl/Total Mercury Concentration Ratio

As a result, the ratio of methyl/total mercury ratio in D laboratory was 90.4% for all, 90.8% for male and 89.8% for female. N laboratory analysis results showed 97.6% for male, 95.4% for female, and 96.8% for all and correlation coefficient of all subjects was 0.1278 (Figure 1(c)). Respectively, N laboratory showed slightly higher value in concentration ratios between the two institutions (p<0.001) (Table 3).

## 4. Discussion

When the results of the two analytical institutions were compared, the total mercury concentration of D laboratory was 0.01 ug/kg higher and the methyl/total mercury ratio of that was 6.5% lower. Between male and female, total mercury concentration in female was higher than that of male, but the ratio of methyl/total mercury was higher in male than female (Table 2). The agreement of concentration in total mercury and methylmercury was high at low concentration of them, but the higher concentration of them, the lower the agreement of analysis results of two institutions. As a result, the difference of methyl/total mercury concentration ratio between the two institutions was high (Figure 1).

Total mercury concentrations in both analytical laboratories were observed at similar levels and the distribution was highly correlated. However, the concentration of methylmercury in N laboratory was much higher than that of D laboratory because of the difference in analytical methods and some concentrations of methylmercury in N laboratory were higher than the total mercury concentration of N laboratory. This is probably due to the difference in the method and recovery rate of total mercury and methylmercury. In the case of methylmercury, the results of CVAFS analysis were lower than those of GC-ECD in both analytical methods, and GC-ECD analysis showed higher levels of methylmercury. However, at 10 ug/kg or less, it is reasonable to conclude that there is no difference in analysis at these low concentrations. The difference between the two methods of analysis is that the differences in the detectors are the most prominent [17, 18]. In this study, some concentration of methylmercury were higher than total mercury concentration in N Lab; it was due to the difference between the analysis methods of methylmercury and total mercury, because methylmercury is a major part of mercury in the blood and the analysis of total mercury does not reflect all of the mercury in the blood. A Korean study found that the ratio of methylmercury to total mercury concentration was 85.1% in students with high total concentrations of blood mercury and 85% and 91% in maternal blood and umbilical cord blood, respectively [19]. In the United States, the ratio of methyl/total mercury concentration was increased with age, and the average ratio of Asian in the US was 0.85, which was close to 0.9 in the 60s. In this study, D laboratory showed similar concentration and N laboratory showed very high results in the 60s as the US study [20].

## 5. Conclusion

This study results was higher or similar to those of previous studies because this is probably due to differences in the analysis subjects. In this study, it was the limitation that the subjects were not able to correct dietary habits and the age of the subjects was high. However, it is very advantageous in that the same elderly study subjects were compared and analyzed quantitatively of methylmercury according to the different analysis methods.

## Figures and Tables

**Table 1:** The analytical conditions of instruments and recovery test result by instruments.

	**Total mercury**	**Methylmercury**
**Recovery test results** **∗**		

**D Lab **%**R(%RSD)**	101 % (0.4%)	106 % (5.0%)

**N Lab **%**R(**%**RSD)**	99 % (2.8%)	101 % (1.4%)

**Methylmercury analytic conditions of instruments**		

**D Lab **	Temperatures of the instruments were 65°C for digestion, 400~500°C for desorption, respectively.	

**N Lab GC conditions**	Temperatures of the injection port, column oven and detector were 180°C, 160°C and 200°C, respectively.	

%R: recovery rate and %RSD: recovery standard deviation. *∗*Both Lab' QC material was CRM (SRM 955c level 3).

**Table 2:** General characteristics and mercury mean concentration of analysis specimens.

	**Total**	**Male**	**Female**
**N (**%**)**	47 (100)	30 (63.8)	17 (36.2)

**Age (years)** **∗**	58.6 ± 1.4	60.8 ± 1.6	54.6 ± 2.7

**D Lab Results**			

**Total mercury (**μ**g/kg)∗∗**	9.5 [7.6, 11.8]	9.0 [6.7, 12.1]	10.5 [7.4, 15.0]

**Methylmercury (**μ**g/kg) ∗∗**	8.6 [7.0, 10.6]	8.2 [6.2, 10.8]	9.4 [6.7 13.3]

**Methyl/total ratio(**%**)**	90.4 [88.9, 91.9]	90.8 [88.8, 92.7]	89.8 [87.4, 92.2]

**N Lab Results**			

**Total mercury (**μ**g/kg) ∗∗**	9.4 [7.4, 11.8]	8.8 [6.5, 12.0]	10.4 [7.2, 15.1]

**Methylmercury (**μ**g/kg) ∗∗**	9.1 [7.2, 11.4]	8.6 [6.3, 11.7]	9.9 [6.8, 14.4]

**Methyl/total ratio(**%**)**	96.8 [95.0, 98.6]	97.6 [96.2, 99.2]	95.4 [91.1, 99.9]

*∗*: mean ± SE (standard error) and *∗∗*: geometric mean [95% confidence intervals]

**Table 3:** Mean difference of mercury mean concentration and methylmercury/total mercury ratio between D Lab and N Lab.

**Categories**	**Total (n-47)**	**Male (n=30)**	**Female (n=17)**
**Total mercury (**μ**g/kg)∗**	-0.01 [-0.14, 0.12]	-0.04 [-0.19, 0.11]	0.05 [- -0.21, 0.30]

**p-value∗∗**	0.898	0.600	0.699

**Methyl mercury (**μ**g/kg)∗**	0.96 [0.69, 1.34]	1.04 [0.48, 1.61]	0.86 [0.18, 1.54]

**p-value∗∗**	<0.001	<0.001	0.017

**Methyl/total ratio (**%**)**	6.5 [4.4, 8.6]	7.2 [5.6, 9.1]	5.2 [ 3.8, 8.1]

**p-value∗∗**	<0.001	<0.001	0.013

*∗*: differences were calculated by N Lab results minus D Lab results, arithmetic mean [95% confidence intervals].

*∗∗*p-values were calculated by one-sample mean comparison test (mean = 0) for difference between D Lab and N Lab.

## Data Availability

The clinical data used to support the findings of this study are available from the corresponding author upon request.
